# Molecular pathogenesis of the myeloproliferative neoplasms

**DOI:** 10.1186/s13045-021-01116-z

**Published:** 2021-06-30

**Authors:** Graeme Greenfield, Mary Frances McMullin, Ken Mills

**Affiliations:** 1 Patrick G Johnston Centre for Cancer Research, Queen’s University Belfast, Belfast, UK; 2 Centre for Medical Education, Queen’s University Belfast, Belfast, UK

**Keywords:** Myeloproliferative neoplasms, Polycythaemia vera, Essential thrombocythaemia, Primary myelofibrosis

## Abstract

The Philadelphia negative myeloproliferative neoplasms (MPN) compromise a heterogeneous group of clonal myeloid stem cell disorders comprising polycythaemia vera, essential thrombocythaemia and primary myelofibrosis. Despite distinct clinical entities, these disorders are linked by morphological similarities and propensity to thrombotic complications and leukaemic transformation. Current therapeutic options are limited in disease-modifying activity with a focus on the prevention of thrombus formation. Constitutive activation of the JAK/STAT signalling pathway is a hallmark of pathogenesis across the disease spectrum with driving mutations in *JAK2*, *CALR* and *MPL* identified in the majority of patients. Co-occurring somatic mutations in genes associated with epigenetic regulation, transcriptional control and splicing of RNA are variably but recurrently identified across the MPN disease spectrum, whilst epigenetic contributors to disease are increasingly recognised. The prognostic implications of one MPN diagnosis may significantly limit life expectancy, whilst another may have limited impact depending on the disease phenotype, genotype and other external factors. The genetic and clinical similarities and differences in these disorders have provided a unique opportunity to understand the relative contributions to MPN, myeloid and cancer biology generally from specific genetic and epigenetic changes. This review provides a comprehensive overview of the molecular pathophysiology of MPN exploring the role of driver mutations, co-occurring mutations, dysregulation of intrinsic cell signalling, epigenetic regulation and genetic predisposing factors highlighting important areas for future consideration.

## Introduction

**Fig. 1:**
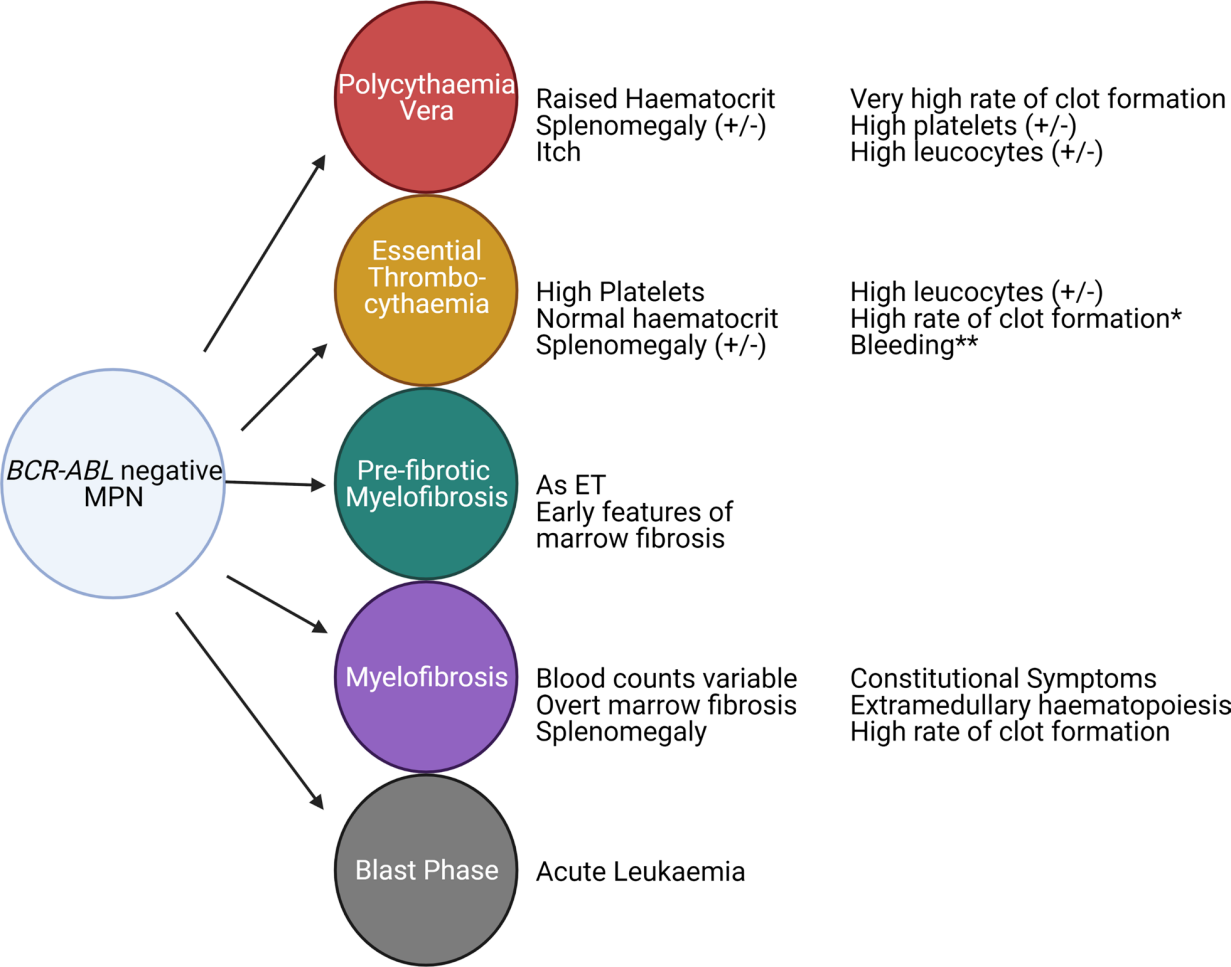
MPN Heterogeneity. A figure demonstrating the distinct clinical entities observed in MPN patients with a summary of distinguishing clinical features observed in each. *Propensity to enhanced rates of clot formation in ET appear to be variable depending on driver mutation status. **Bleeding can manifest in a minority of ET patients resulting from acquired Von Willebrand disease resulting from very high platelet counts

The classical Philadelphia chromosome negative myeloproliferative neoplasms (MPN) are rare clonal neoplastic disorders of the myeloid haematopoietic stem cells (HSC). These disorders are classified into polycythaemia vera (PV) with a predominance of excessive red cell production, essential thrombocythaemia (ET) with a predominance of excessive platelet production and primary myelofibrosis (PMF) with excessive bone marrow scarring and fibrosis. The updated WHO classification also includes pre-fibrotic myelofibrosis (Pre-PMF), distinguishing a group of patients with subtle phenotypic differences from ET and a higher rate of progression to myelofibrosis (MF) [1]. Prognosis is highly variable, but in general, MF significantly limits life expectancy in comparison to PV or ET. A small number of individuals progress to blast phase of disease presenting as acute myeloid leukaemia which is frequently refractory to conventional therapy. Figure 1 characterises the distinguishing clinical features commonly observed in each MPN manifestation.

Despite the obvious differences, similarities in bone marrow morphology, a tendency to arterial and venous thrombus formation and a tendency to secondary myelofibrotic or leukaemic phase transformation links these disorders clinically. These phenotypic similarities had been identified well in advance of the discovery of activating mutations in the *JAK2*, *MPL* and *CALR* genes and the demonstration of activated Janus Kinase (JAK)/signal transducer and activator (STAT) signalling pathway signalling which has helped to further define these disorders [2–8]. *JAK2* V617F mutations are detectable in approximately 95% of PV patients with *JAK2* exon 12 mutations present in virtually all remaining PV cases [3–5]. The *JAK2* V617F mutation is present in approximately 50% of ET and PMF patients with *CALR* and *MPL* mutations present in most remaining patients [8, 9]. “Triple negative” patients make up a small percentage of ET and PMF cases. Diagnostic criteria now place a heavy emphasis on demonstrating the presence of these genetic changes to confirm a suspected diagnosis as is demonstrated in Table 1 [10].

**Table 1:** A summary of the 2016 World Health Organisation (WHO) diagnostic criteria

Disease	Major criteria	Minor criteria	Diagnosis	Reference
Polycythaemia vera	1. Hb > 16.5 g/dL (M) 16.0 g/dL (F) or, haematocrit > 49% (M) 48% (Female) or, increased red cell mass (> 125%)^a^ 2. Bone marrow biopsy with characteristic morphology 3. Presence of *JAK2* V617F or *JAK2* exon 12 mutation	1. Serum erythropoietin level below normal	All 3 major criteria or, Top 2 major and the minor criteria	[1]
Essential Thrombocythaemia	1. Platelet count > 450 × 10^9^/L 2. Bone marrow biopsy with characteristic morphology 3. Not meeting criteria for another MPN/myeloid neoplasm 4. Presence of *JAK2*, *CALR,* or *MPL*	1. Presence of another clonal marker or absence of evidence for a reactive thrombocytosis	All 4 major criteria or, Top 3 major and the minor criteria	[1]
Pre-fibrotic primary myelofibrosis	1. Bone marrow biopsy with characteristic morphology without reticulin fibrosis > grade 1 2. Not meeting criteria for another MPN/myeloid neoplasm 3. Presence of *JAK2*, *CALR* or *MPL* mutation, or, another clonal marker, or, no identifiable cause of reactive fibrosis	1. Anaemia not caused by a co-morbid condition 2. Leukocytosis ≥ 11 × 10^9^/L 3. Palpable Splenomegaly 4. Lactate dehydrogenase above upper limit of normal	All 3 major criteria plus at least one minor criteria (confirmed on two separate measurements)	[1]
Myelofibrosis	1. Bone marrow biopsy with characteristic morphology with either reticulin or collagen fibrosis grades 2 or 3 2. Not meeting criteria for another MPN/myeloid neoplasm 3. Presence of *JAK2*, *CALR* or *MPL* mutation, or, another clonal marker, or, no identifiable cause of reactive fibrosis	1. Anaemia not caused by a co-morbid condition 2. Leukocytosis ≥ 11 × 10^9^/L 3. Palpable Splenomegaly 4. Lactate dehydrogenase above upper limit of normal 5. Leukoerythroblastosis	All 3 major criteria plus at least one minor criteria (confirmed on two separate measurements)	[1]
Blast phase MPN	Patients with MPN and peripheral or bone marrow myeloid blast percentage > 20%		Major criteria met	[11]

A summary of the 2016 WHO criteria for the distinct clinical entities of PV, ET, Pre-PMF, PMF. Consensus diagnostic criteria for blast phase transformation are included. A minority of patients with a diagnosis of a MPN disorder may not meet diagnostic criteria for any of these distinct entities of any other myeloid neoplasm and may be classed as MPN unclassifiable [1]

^a^British Society of Haematology guidelines propose higher haematocrit levels of > 52% in males and > 48% in females [12]

With modern diagnostic approaches, it is increasingly clear that the desire for neat classification is often complicated by a spectrum of phenotypic presentation and genetic heterogeneity. A range of co-occurring somatic mutations are frequently detectable at significant variant allele frequencies alongside the *JAK2*, *MPL* or *CALR* mutations [13–15]. Complex clonal hierarchies have been observed within MPN patients [16]. These frequently observed co-occurring mutations include genes encoding epigenetic modifiers, transcriptional regulators and mRNA splicing machinery. They are not exclusive to MPN but rather occur across the spectrum of myeloid malignancy [17, 18]. Further complicating the picture, many of these mutations, including *JAK2* V617F, are increasingly detected in individuals as we age yet with the majority demonstrating no haematological disease phenotype [19]. This clonal haematopoiesis of indeterminate potential (CHIP) unsurprisingly pre-disposes to the development of myeloid malignancy but appears to also be sufficient to significantly increase cardiovascular risk [19–21].

Current therapeutic approaches in MPN aim to limit the risk of thrombosis with antiplatelet agents, anticoagulants, therapeutic venesection and cytoreductive therapies including hydroxycarbamide and interferon-alpha all with established benefits in specific circumstances [22–24]. JAK inhibitors including ruxolitinib have provided an additional targeted therapy with clear symptomatic and clinical benefits but limited disease-modifying activity [25–28]. Haematopoietic stem cell transplantation offers the only opportunity for cure but is rarely suitable due to the significant toxicities and mortality risk associated. It is generally reserved for younger, fitter individuals with higher-risk myelofibrosis or blast phase disease, and outcomes remain poor in these populations [29, 30].

The clinical and genetic similarities and differences in this heterogeneous population offer the opportunity to characterise and elucidate the contributions of various genetic and epigenetic factors to disease pathogenesis. Enhanced availability of such genetic and phenotypic data has meanwhile provided the opportunity to generate individualised prognosis probabilities to MPN patients [15]. This review of current understanding of the molecular pathogenesis of MPN will focus on the role of JAK/STAT and other intracellular signalling pathways, acquired and inherited genetic contributors to disease, epigenetic dysregulation and cellular context and will highlight areas for future research considerations.

## JAK/STAT signalling in MPN

The evolutionarily conserved JAK/STAT pathway exists as a critical intracellular mediator of extracellular protein–cell surface receptor interactions. Four genes for JAK proteins exist in the human genome (*JAK1*, *JAK2*, *JAK3* and *TYK2*) interacting with seven STAT proteins to mediate differential effects on transcriptional control. JAK proteins associate with numerous cell surface receptors, and thus, JAK/STAT signalling cascades are activated in many metabolic functions, immune cell functions and control of haematopoiesis [31]. Effective control of erythropoiesis, megakaryopoiesis and granulopoiesis is essential to respond to changing physiological demands throughout life and in times of physiological stress or infection. Hormonal signalling with erythropoietin (EPO), thrombopoietin (TPO) and granulocyte-colony stimulating factor (GCSF) drive enhanced production of red cells, platelets and granulocytes through the respective receptors. Activation of the erythropoietin receptor (EPOR), thrombopoietin receptor (MPL) and granulocyte-colony stimulating factor receptor (G-CSFR) then activate JAK/STAT pathways to drive proliferation. JAK/STAT signalling is heavily interconnected with many core cancer signalling pathways and cellular functions including metabolism, cell cycle control, apoptosis, DNA damage response and direct or indirect transcriptional control [32]. Abnormal JAK/STAT signalling has been implicated across a range of myeloid, B and T lymphoid haematological malignancies and solid tumours [33–36].

### Driver mutations activating JAK/STAT signalling

In MPN, constitutive activation of the JAK/STAT signalling pathway is a critical mediator of the pathogenesis. A point mutation in exon 14 of the *JAK2* gene results in a single amino acid (valine to phenylalanine) substitution and conformational change in the JH2 pseudo-kinase domain of JAK2. This results in constitutive tyrosine phosphorylation activity by disrupting the normal inhibitory action of the JH2 domain. This *JAK2* V617F transcript therefore drives constitutive activation of the JAK/STAT pathway in the absence of EPOR, MPL or G-CSFR ligand binding. This mutation is detected in 95% of PV patients and approximately 50% of ET and PMF patients [5, 37, 38]. The resulting disease phenotype is subject to several additional variables including homo or heterozygosity of the *JAK2* V617F, variant allele frequency, additional co-operating mutations and/or external influences including iron deficiency. The remaining 5% of PV patients are almost entirely accounted for by mutations in exon 12 of the *JAK2* gene through predominant activation of EPOR signalling pathways driving an erythrocytosis [4]. These *JAK2* exon 12 mutations have not been seen in PMF or ET.

The majority of *JAK2* V617F negative ET and PMF patients have detectable mutations in *MPL* or *CALR* [7, 8, 38]. These mutations drive disease through activation of MPL receptor and subsequent downstream JAK/STAT activation. Generally, the driver mutations occur in a mutually exclusive manner. A number of activating *MPL* mutations have been identified in the transmembrane domain encoded by exon 10 in both familial and sporadic forms of MPN [8, 39]. These gains of function mutations including W515L and S505N constitutively activate downstream JAK/STAT signalling by removing an inhibitory element and inducing dimerization, respectively [39, 40]. Additional activating or augmenting mutations identified in *MPL* transmembrane domain by deep mutational scanning screens have also been previously identified in MPN patients demonstrating an inherent susceptibility in the *MPL* gene [41].

Calreticulin (CALR) is an endoplasmic reticulum chaperone protein which in mutant form will interact directly with the TPO receptor MPL driving TPO independent activation. Numerous mutations in the *CALR* gene have been described with the majority classed as type 1 resulting from a 52-bp deletion in exon 9 or type 2 with a 5-bp insertion in exon 9. The subsequent activation of MPL and downstream JAK/STAT signalling is dependent on a positively charged C terminus resulting from a frameshift in exon 9 and enabling the CALR lectin binding domain to maintain a stable interaction with MPL [42]. There is a recognised distinct clinical phenotype between patients with detectable type 1 and type 2 *CALR* mutations in both patients and murine models. Type 1 deletions are significantly over-represented in myelofibrosis and produce a more pronounced MPN phenotype in mice [43].

A small minority of ET and PMF patients fall into the “triple negative” category with no detectable mutation in *JAK2*, *MPL* or *CALR.* The incorporation of additional genetic tests into the work-up of these patients has challenged the notion of true triple negativity in MPN. Retrospective evaluations demonstrate that some of these patients may have other genetic markers of clonality detectable or subsequently test positive for a driver mutation. A few patients remain with characteristic phenotypic and morphological features and no detectable genetic abnormalities [44].

Frequently, these JAK/STAT activating driver mutations are the only detected genetic abnormality in MPN patients with one large study reporting this in 45% of patients using a targeted myeloid next-generation sequencing (NGS) panel [15]. The presence of *JAK2*, *CALR* or *MPL* mutations alone are sufficient to generate an MPN phenotype, albeit polyclonal in nature, in murine models [45]. One study of gene expression profiling by microarray analysis demonstrated features of activated JAK/STAT signalling in MPN patients regardless of clinical phenotype or mutational status [2]. Ruxolitinib, a JAK1/2 inhibitor, is effective across all mutant driver backgrounds [46]. It is therefore clear that constitutively activated JAK/STAT signalling is a key feature of disease pathogenesis.

The presence of the *JAK2* V617F mutation is also evidently more than a simple switch for excess proliferation. There is significant heterogeneity in terms of the variant allele frequency (VAF), and therefore, clonal size measured in peripheral blood granulocytes is detectable across the MPN patient population [47]. Patients with homozygosity or high VAF tend towards a PV phenotype rather than ET [48, 49]. And yet, there are many PV patients with a low VAF and similarly ET patients with high VAF. A rare subgroup of patients presenting with splanchnic vein thrombosis (SVT) frequently exhibit normal or near normal blood counts with a small *JAK2* V617F clone detectable [50]. The *JAK2* V617F mutation is also detectable in individuals with CHIP who exhibit no MPN phenotype. Despite the normal blood counts, these individuals have been observed to have a significantly increased risk of cardiovascular disease [20]. Paradoxically, *JAK2* V617F positive ET cases are significantly more likely to have thrombotic complications despite lower platelet counts than their *CALR* mutated comparators [51]. One suggestion in these cases is that the mutant JAK2 results in qualitative changes enhancing the pro-thrombotic phenotype. There is evidence to suggest enhancing endothelial–erythrocyte interactions via activation of Lu/BCAM or enhanced neutrophil extracellular trap formation [52, 53]. A recent study has demonstrated that in patients with a low VAF (< 20%) and therefore small mutant clones within the peripheral blood granulocytes, there is significant heterogeneity of clone size within the reticulocytes and platelets measured using a quantitative polymerase chain reaction established to measure *JAK2* V617F RNA. In many cases, the clonal sizes in the reticulocytes and platelet populations were much higher than the granulocytes perhaps from late expansion of erythroid and platelet precursors [54]. We may not therefore have been accurately assessing clonal size in many of our “low allele” patients, and granulocyte VAF may underestimate the qualitative effect of the mutant JAK2 presence.

### STAT proteins in MPN

The complexity of STAT signalling has identified roles for STAT proteins in oncogenesis and tumour suppression, occasionally with conflicting roles in the same tumour type [55]. Investigations of STAT protein recruitment, phosphorylation and ultimately dominance of transcriptional control in MPN have focused on the role of STAT5, STAT1 and STAT3. STAT5 activation was identified early as a key mediator of MPN pathogenesis with experimental work able to demonstrate a dependence on STAT5 to generate a MPN phenotype [56, 57]. STAT5 transcriptional activity is upregulated by the expression of *JAK2* V617F in cell lines [37]. In analysis of ex vivo colony forming assays from ET and PV patients, transcriptional analysis demonstrated an enrichment of STAT5A targets with nuclear phosphorylation of STAT5A identified in *JAK2* V617F position colonies from both ET and PV patients but not wild type colonies whilst a recent phospho-proteomics approach identified STAT5 and STAT3 as differentially phosphorylated in *JAK2* V617F mouse haematopoietic cells [58, 59]. Conditional expression of a null *STAT5a/b* gene resulted in a failure of a *JAK2* V617F mouse model to develop polycythaemia but did not abrogate the risk of myelofibrosis [60]. In an alternative *JAK2* V617F mouse model STAT5 deletion resulted in loss of the PV phenotype which could be rescued by STAT5 re-expression [61]. Taken together, STAT5 signalling appears to play a key mediator role in generating the PV phenotype.

Identification of enhanced enrichment of Interferon-gamma target genes in ET in comparison to PV highlighted STAT1 signalling as a potential mediator of differential molecular response between the two disorders. In keeping with this, phosphorylated STAT1 was detectable in ET patients and not PV patients ex vivo [58]. Murine models subsequently demonstrated the loss of STAT1 producing a phenotype favouring erythropoiesis at the expense of megakaryopoiesis and with a reduction in fibrosis [62]. STAT1 phosphorylation at serine 727 may drive proliferation and restrain megakaryocyte differentiation in blast phase MPN. Blocking this serine phosphorylation resulted in different functional outcomes in comparison to blocking tyrosine phosphorylation with ruxolitinib [63]. These results have suggested that an altered balance between STAT1 and STAT5 signalling may be one possible cell intrinsic mechanism of phenotype determination. However, erythroblasts harbouring the *JAK2* exon 12 mutations which drive erythrocytosis and are only associated with PV have a transcriptional profile which cannot be distinguished from *JAK2* V617F positive ET erythroblasts with no differential in STAT1 activation [64].

Constitutive activation of STAT3 was identified in a number of MPN patients from granulocytes in advance of the discovery of *JAK2* V617F, whilst higher levels of STAT3 tyrosine phosphorylation have been identified in *JAK2* V617F positive individuals and as a result of *JAK2* V617F expression in murine models [65–67]. A murine model of STAT3 hyperactivity induced by deletion of suppressor of cytokine signalling (SOCS) 3 spontaneously develops myeloproliferative and lymphoproliferative pathology with serine phosphorylation of STAT3 critical [68]. STAT3 deletion results in an altered MPN phenotype in *JAK2* V617F mice with reduced neutrophilia and enhanced thrombocytosis present [69].

The canonical tyrosine phosphorylation, nuclear translocation and transcription factor activity are only one role of the STAT proteins. Unphosphorylated STAT proteins appear to have important roles in the normal maintenance of the epigenome in HSC and progenitor cells [70]. As mentioned, serine phosphorylation of STATs may affect transcriptional control and the methylation of STAT3 by Enhancer of Zeste 2 (EZH2) identified as a mediator of transcriptional control in other solid tumours requires investigation in MPN to fully understand the dynamics at play between STAT proteins and final phenotypes [63, 68, 71]. Therefore, as with all aspects of molecular biology in MPN, differential STAT1/STAT3/STAT5 mobilisation is likely to tell only part of the story.

## Non-JAK/STAT signalling in MPN

Outside of JAK/STAT signalling, it is increasingly evident that activation of STAT independent phosphoinositide 3-kinase (PI3K) and mitogen-activated protein kinase (MAPK) signalling pathways is important in the disease pathogenesis of *JAK2* V617F positive MPN [37]. *CALR* mutations have also been observed to activate MAPK signalling pathways albeit with a differential expression profile evident in comparison to *JAK2* V617F [72, 73]. There is evidence that either or both signalling of these pathways may remain active in the presence of the JAK inhibitor ruxolitinib. Murine MPN models with *JAK2* V617F and *MPL* W515L drivers have demonstrated persistent activation of the MAPK mediated by platelet-derived growth factor receptor alpha (PDGFRα) in vivo in the setting of ruxolitinib exposure. Combined JAK/MEK inhibition in this model was more efficacious [74]. Persisting phosphorylation of serine residues on STAT5B has been observed in the *JAK2* V617F positive SET2 cell line model dependent on PI3K/mechanistic target of rapamycin (mTOR) activation with enhanced efficacy again observed when combining JAK inhibition with either PI3K or mTOR inhibitors [75]. Early phase trials of everolimus, an mTOR inhibitor, in MF patients have previously demonstrated some clinical benefit [76]. Activated MAPK, PI3K/AKT and JAK/STAT signalling are also observed in numerous myeloid malignant phenotypes including acute myeloid leukaemia (AML), chronic myeloid leukaemia (CML), atypical CML, chronic myelomonocytic leukaemia and juvenile myelomonocytic leukaemia [77]. Clearly, improving our understanding of the intricacies of dysregulated signalling cascade activation in MPN patients and the effect of treatment may offer some opportunity to manipulate these processes for more efficacious treatments in select individuals.

### Negative regulation of intracellular signalling in MPN

In normal health, intracellular signalling cascades are closely regulated positively by ligand binding to cell surface receptors and negatively by a number of intracellular components acting predominantly as phosphatases or targeting proteins for ubiquitination and subsequent proteasome-mediated degradation. The SOCS proteins are critical negative regulators for JAK/STAT signalling. SOCS3 is a key negative regulator of EPO signalling and therefore erythropoiesis through its interaction with JAK2 and EPOR. There is conflicting evidence on the role of SOCS proteins in the regulation of mutant JAK2. One study has demonstrated that in the presence of the *JAK2* V617F mutant, SOCS3 undergoes tyrosine phosphorylation, losing the ability to negatively regulate JAK2 and may in fact potentiate the effect by stabilising the mutant JAK2 [78]. A more recent investigation has demonstrated that mutant JAK2 exhibits comparable inhibition by SOCS3 in in vitro kinase assays [79]. Knockdown of SOCS3 in *JAK2* V617F expressing Ba/F3 cells further enhances phosphorylation of STAT3, STAT5 and extracellular signal-regulated kinase 1/2 (ERK1/2) suggesting a tumour suppressor role for SOCS3 in the mutant state [80]. One small study has suggested aberrant regulation of SOCS3 expression in PMF with observation that SOCS3 promoter regions were methylated in 32% of PMF patients but not ET or PV patients [81]. The JAK inhibitor ruxolitinib can clearly reduce the expression of SOCS3 in a number of cellular contexts outside of MPN [82, 83] and we and others have shown that RNA sequencing data confirm reduced expression in MPN cell line models [84, 85]. Histone deacetylase inhibition may upregulate SOCS3 expression in MPN [86–88].

*CBL* encodes the E3 ubiquitin ligase Cbl proto-oncogene (CBL) and is an important regulator of many tyrosine kinases. *CBL* mutations have been identified in many malignancies and at a low but significant level in MPN [15]. These mutations drive myeloproliferation and result in activated JAK/STAT and PI3K/AKT signalling in murine models [89]. In addition, loss of specific phosphatase activity of phosphatase and tension homolog deleted on chromosome 10 (PTEN), a regulator of PI3K/AKT signalling can drive an MPN phenotype [90]. Conversely, higher expression of dual-specificity phosphatase 1 (DUSP1) may be required in *the JAK2* V617F context to protect the cells by moderating JNK/P38 MAPK signalling and protecting against accumulating DNA damage [91].

**Fig. 2:**
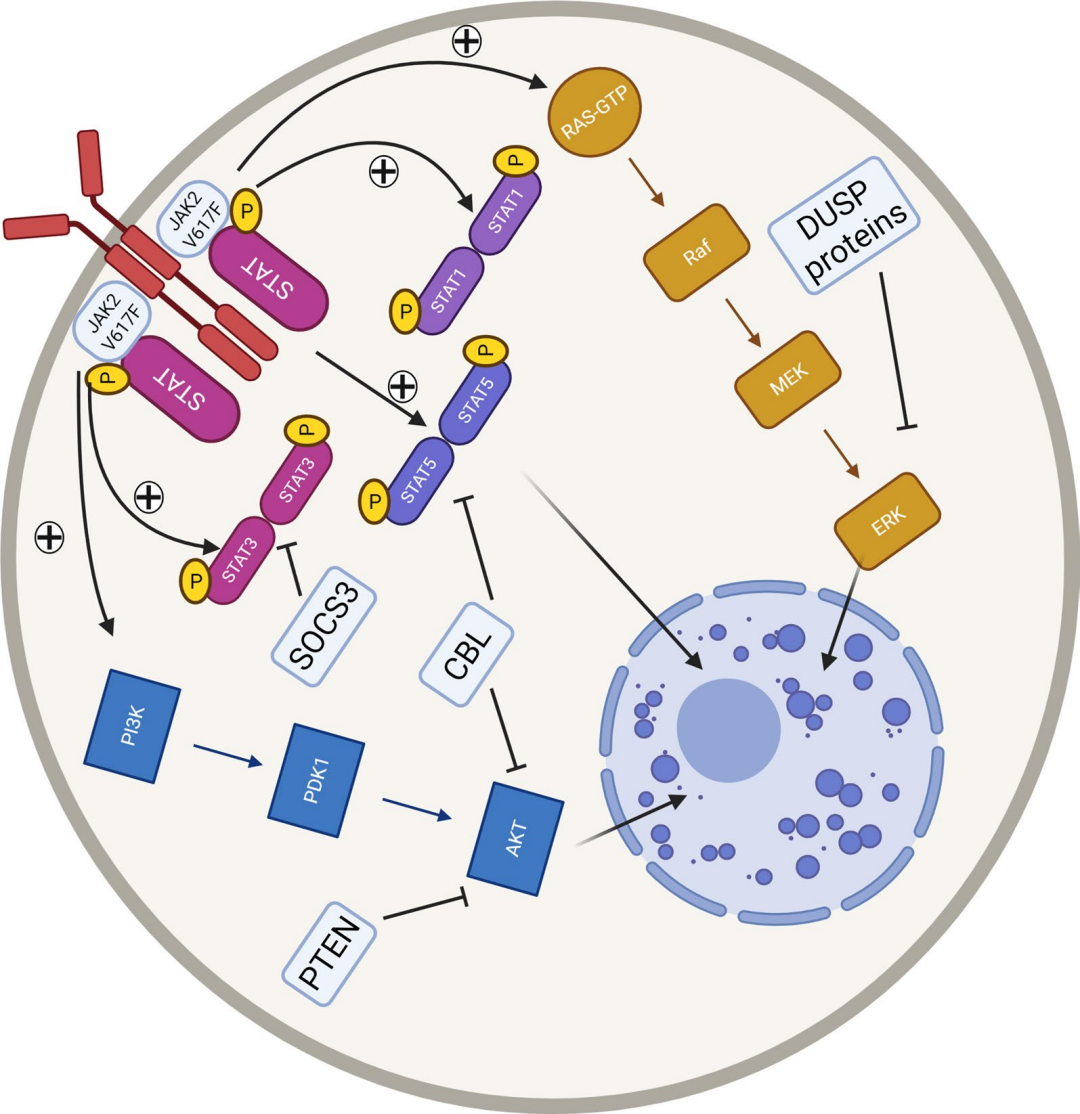
Activated signalling in MPN. Activated signalling pathways in MPN include JAK/STAT signalling, PI3K/AKT and MAPK (RAS/MEK/ERK) cascades. The balance of predominant STAT1/3/5 signalling may impact on ultimate disease phenotype, whilst persistence of activated MAPK or PI3K signalling or STAT serine phosphorylation may occur despite JAK inhibitor therapy. Examples of intracellular negative regulators of JAK/STAT, PI3K/AKT and MAPK signalling with potential roles in MPN pathophysiology are also shown

Figure 2 highlights active intracellular signalling cascades identified in MPN and examples of intracellular negative feedback mechanisms. Understanding the complexities of this intrinsic regulation of intracellular signalling in vivo in the heterogenous cellular contexts of MPN patients may help to improve our understanding of pathogenesis and treatment options.

## Co-occurring mutations and clonal evolution in MPN

With the increasing availability of genetic sequencing in the research and now routine diagnostic setting, the genetic heterogeneity of the MPN group has become increasing clear. A number of pathological mutations are frequently and recurrently identified in MPN patients across a range of genes affecting epigenetic regulation, transcriptional control and splicing machine. These genes commonly include *ASXL1*, *DNMT3A* and *TET2* at relatively high frequencies in upwards of 5% of patient samples across the MPN spectrum. Others including *CBL*, *SF3B1*, *EZH2*, *TP53*, *SRSF2*, *USAF1* and *IDH1/2* are identified in fewer than 2% of patients in large studies [15]. These mutations are regularly identified in other individuals across the range of myeloid malignancy and in the CHIP population [92–94]. These mutations which have been well characterised represent only a small proportion of the overall mutational burden seen as aging progresses in MPN patients when whole genome sequencing approaches are employed [95].

### Prognostic implications

**Fig. 3:**
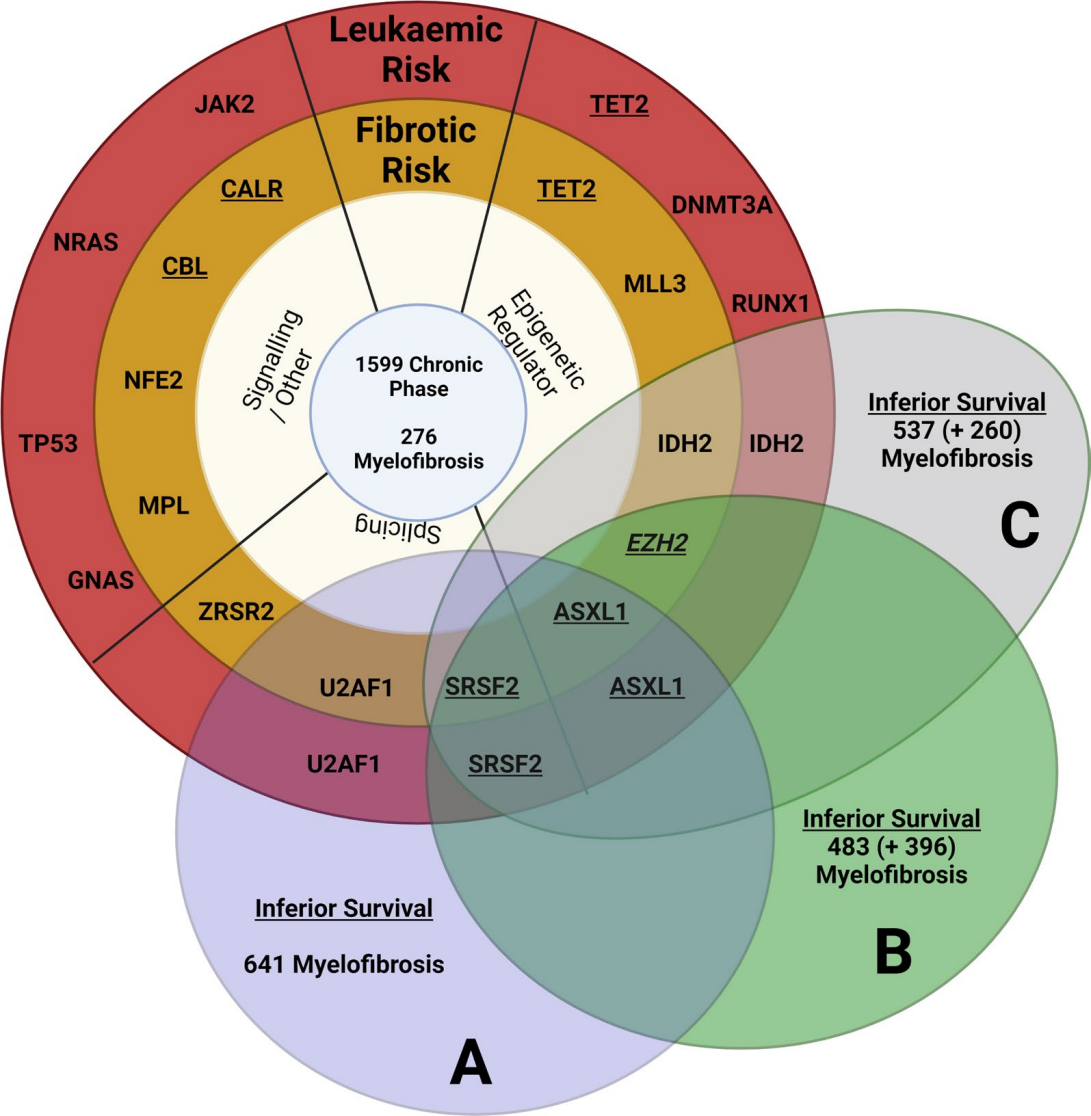
Mutational significance in MPN for overall survival. A summary of determined mutational significance in MPN. The central figure shows genetic contributors to fibrotic (inner yellow ring) and leukaemic (outer red ring) transformation in MPN determined by a predictive modelling approach in 1599 chronic phase and 276 myelofibrosis patients [15]. Underlined genes were contributors to death in myelofibrosis patients in this modelling. Overlaid are a purple ring** (A)** showing independent risk factors for survival identified in 641 myelofibrosis patients [101], a green ring** (B)** showing independent risk factors for survival identified in 483 myelofibrosis patients and validated in a further 396 patients [98], and finally a grey ring** (C)** showing prognostically detrimental genes in 537 myelofibrosis patients and validated in a further 260 patients [102]

As larger cohorts of individuals continue to be analysed it is clear that the presence of particular mutations is associated both with the disease phenotype and ultimate prognosis. Chronic phase PV and ET patients are significantly more likely to have no additional mutations when compared to PMF patients. The occurrence of particular genes has been significantly associated with disease phenotype. For example, *NFE2* mutations correlate with a PV phenotype, spliceosome component mutants and epigenetic regulators *EZH2* and *ASXL1* are more frequently observed in PMF, whilst other genes including *IKZF1* are almost exclusively observed in blast phase disease [15, 96, 97]. Early studies demonstrated clear prognostic implications of particular co-operating mutations. In a cohort of 483 European PMF patients with validation in an American group, *ASXL1*, *EZH2* and *SRSF2* mutations independently predicted shortened survival. *IDH1/2* or *SFSF2* mutations were associated with leukaemic progression in these cohorts with *TP53* strongly associated in another cohort [98, 99]. The negative prognostic impact of *ASXL1* appears to be evident when present with another high risk mutation but not solely on its own [100]. In a large study of 2035 patients, eight genomic subgroups were identified within the MPN spectrum, each reflecting a different proportion of PV, ET and PMF patients with variable risks of leukaemic or fibrotic transformation and overall survival. Genetic factors including *TET2*, *SRSF2* and *ASXL1* mutations contributed to over 50% of the risk factors for fibrotic transformation from PV or ET using a predictive modelling approach. Similarly, over a third of the risk of leukaemic transformation was attributable to genetic factors including *TP53* mutation [15]. Figure 3 demonstrates mutated genes identified as contributors to leukaemic and fibrotic transformation from this predictive modelling approach integrated with results from multivariate analysis approaches in other large cohorts [15, 98, 101, 102]. Individualised prognostic evaluations can now be achieved at diagnosis based on the incorporation of genetic and clinical information, and molecular data are now routinely incorporated into prognostic scoring systems including MIPPS70 + and GIPSS [101, 103].

### Clonal structure and evolution

The variant allele frequency reported by next-generation sequencing (NGS) myeloid panels provides a rough estimate of clonal size and dominance, reflecting both the number of affected cells and whether these cells are hetero or homozygous for the mutation of interest. Variant allele frequencies of driver and co-occurring mutations are seldom identical, reflecting a clonal hierarchy comprising dominant and sub-clones with differing genetic abnormalities and resulting competitive advantages. Application of single-cell technologies in myeloproliferative patients has demonstrated complex clonal hierarchies with dominant and sub-clones demonstrating distinctive transcriptomic signatures within individual patients [16]. The clonal complexity is greater in MPN in comparison to CHIP patients but much less than that observed in AML [104].

This complexity reflects clonal evolution over time, occurring as cells acquire additional genetic or epigenetic changes driving further divergence from the cell of origin. This can significantly alter the clonal structure of the disease with smaller sub-clones developing the competitive advantage to establish dominance. Patients progressing to myelofibrosis and AML often demonstrate greater genetic complexity within clones with increasing numbers of mutations detectable. Ultimately, this results in transcriptional and functional changes which are observed in patients cells underlying the progressive disease phenotype [105]. In chronic phase MPN, the rate of acquisition of new mutations is generally considered to be a slow process. Only two additional mutations were observed over the equivalent of greater than 130 patient years in one study [99]. However, this generalisation, like many, does not reflect the significant heterogeneity across the MPN patient spectrum with both acquisition of new clones resulting from new mutations or minor sub-clones establishing dominance observed in patients transforming from chronic phase to blast phase disease [104, 106]. Meanwhile, approximately one-third of PMF individuals receiving the JAK inhibitor ruxolitinib demonstrated clonal evolution on therapy acquiring new mutations [107]. Perhaps as our repositories of sequential pre- and post-MPN progression sequencing data increases, we may understand the genetic, epigenetic and extrinsic factors explaining the propensity for clonal evolution in a number of individuals.

Recent comprehensive genomic profiling with a whole genome sequencing approach and phylogenetic reconstruction based on the numerous somatic mutations occurring during life have provided evidence that acquisition of both driver and common co-occurring mutations may occur many decades prior to disease presentation. In a number of cases, the acquisition of *JAK2* V617F or *DNMT3A* mutations were predicted to have occurred in utero*.* This approach reveals a significant variability in the rates of clonal expansion, dependent on the mutational landscape of the clone [95]. A concept of mutational compatibility can be theorised from much of the genetic data available in myeloid malignancy to date. A number of individual mutations are frequently observed together, whilst others appear strictly mutually exclusive of each other [99]. In some cases, this mutational compatibility clearly drives a competitive advantage for the cell such as that demonstrated by *FLT3-ITD* and *NPM1* mutations in AML, rapidly establishing a clonal dominance and driving a clear phenotype [104]. In MPN, this mutational compatibility is more subtle but evident from the high frequencies of many mutations including *TET2*, *ASXL1* and *DNMT3A* occurring alongside drivers observed across the MPN patient spectrum. It is interesting that many of these co-occurring mutations have been observed to be the initiating mutation prior to the acquisition of one of the classical driver mutations or may occur subsequently to it [108, 109]. This bi-directional co-occurrence suggests more than a simple random coincidence in the acquisition of these mutations.

### Mutational order

**Fig. 4:**
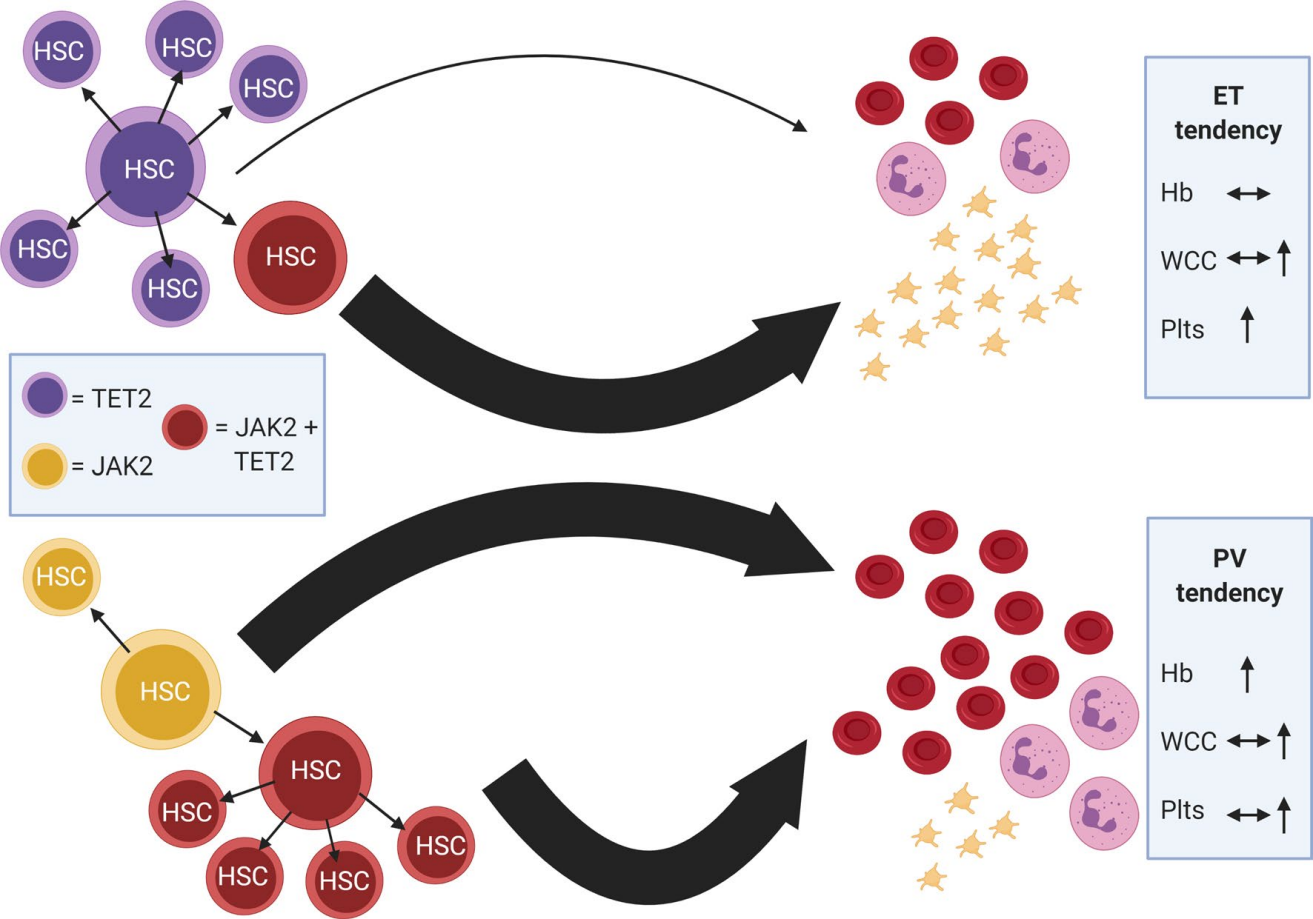
Effects of mutation order in MPN. Order of mutation acquisition affects both self-renewal capacity of mutants in the haematopoietic stem cell compartment and proliferative drive ultimately affecting the probability of producing a PV or ET phenotype

Acquisition order also appears to be a critical determinant of the resulting disease phenotype. Order of acquisition can be inferred from colony assays or single-cell studies allowing genotyping of the clonal structure. In the case of *TET2*, it is evident that HSCs with this mutation are transcriptionally altered, driving expansion of the clone within the HSC compartment but with limited excess production of terminally differentiated megakaryocytes or erythrocytes until a second hit with the *JAK2* V617F mutation occurs. In contrast, *JAK2* V617F first cells behave in precisely the opposite manner [108]. Consistent with these findings, loss of *TET2* has been observed to promote clonal expansion and self-renewal of HSCs in murine models [110, 111]. *TET2-*first individuals tend towards an ET phenotype with higher numbers of single mutant cells in the HSC compartment. In contrast, *TET2-second* individuals show a predominance of double mutant cells in the HSC compartment with a tendency to PV [108]. Figure 4 demonstrates this pictorially. Similarly, loss of function *DNMT3A* mutations appears to enhance the self-renewal of HSCs and *DNMT3A* null HSCs are serially transplantable to a significantly enhanced degree in comparison to normal HSCs [112, 113]. Loss of function of another epigenetic regulator *EZH2* has also been observed to enhance self-renewal of the *JAK2* V617F positive HSC in a murine model [114]. *JAK2* V617F only clones in contrast do not promote an MPN disease phenotype on serial transplantation [111]. Early observations that *JAK2* V617F positive individuals could transform to *JAK2* V671F negative blast phase disease appear to highlight the ability of pre-*JAK2* clone with self-renewing capacity in the stem compartment to later establish dominance over the *JAK2* clone [115]. Recent advances have moved away from a traditional model of haematopoiesis in which precursors progress through a series of discrete intermediate stages with decreasing differential potential at each stage to a continuum where the boundaries between stem and progenitor cells are increasingly blurred [116]. The epigenetic regulation of transcriptional control affected by loss of normal *TET2* and *DNMT3A* may allow HSC to access alternative transcriptional programmes, shift along this continuum and promote self-renewal. It is interesting that in CHIP individuals, *TET2* and *DNMT3A* clonal fractions are approximately 25% and 14% smaller than *ASXL1* clonal fractions suggesting a differential ability to promote clonal expansion [117]. In any instance, it is clear that the field in which the *JAK2* mutation is sown is very different in the context of an earlier mutation. Whether these *TET2* or *DNMT3A* mutant first cells are primed for *JAK2* mutagenesis or the subsequent frequency of *JAK2* V617F mutations simply reflects random chance and high turnover is not clear at this stage.

### Cell of origin

Further cellular context is provided by the cell of origin and ultimate differentiation bias of this cell. Detection of driver mutations in multiple different mature cell compartments including erythroid, megakaryocyte, granulocyte, monocyte and in some cases lymphoid cells provide evidence of an initiating cell with multipotency implicating the HSC population [118]. In keeping with this, *JAK2* and *CALR* mutants are detectable within CD34+, CD38- HSC and CD34+, CD38+ progenitor populations [119]. A murine model incorporating a humanised ossicle niche has demonstrated engraftment of myelofibrosis from the CD34+, CD38− HSC population only, with no engraftment in the CD34+, CD38+ progenitor population, suggesting that the initiating cell resides within this HSC population [120]. A knock-in *JAK2* V617F model has also demonstrated the critical role of the HSC population in initiation and maintenance of the polycythaemia phenotype [121]. Therefore, involvement of the multi-potent HSC population appears to be important.

Yet, multipotency of these cells in experimental conditions may not reflect the true balances and equilibriums at play in a complex multi-factorial environment such as the bone marrow. In fact, some of these cells appear to have an in-built differentiation bias from a very early position in the haematopoietic hierarchy. In mouse models, platelet biased HSC populations have been identified at the apex of the hierarchy, with enhanced capability of short- and stable long-term platelet production without loss of self-renewal ability and retain the ability in a proportionally limited manner give rise to lymphoid biased HSCs [122]. It is not clear if HSC exist with other lineage bias [123]. Application of single-cell technologies has demonstrated this megakaryocyte differentiation bias in HSCs in myelofibrosis patients with the majority of megakaryocyte progenitors transcriptionally distinct from normal, with proliferative and fibrosis enhancing gene signatures evident [124]. Similarly, single-cell studies in *JAK2* V617F positive ET patients have demonstrated an expanded population of megakaryocyte primed HSCs with increased sensitivity to interferon alpha signalling [125]. These findings point towards an inherent bias in phenotype, at least towards ET or PMF, depending on the bias on the initiating stem cell acquiring the first mutation.

The interaction between the malignant clone and stromal cells by means of pro-inflammatory and cytokine signalling is a key determinant in the formation of a fibrotic phenotype. Myofibroblasts responsible for the deposition of collagen are derived from multipotent mesenchymal progenitor stromal cells [126–128]. These myofibroblasts evolve over time, undergoing a maldifferentiation process to lose ability to support haematopoietic tissue and contribute to marrow fibrosis. The presence of the malignant haematopoietic clone and resulting inflammatory milieu provide a continuous drive for myofibroblast differentiation and vicious cycle of fibrosis [128]. Removing Gli1^+^ mesenchymal stromal cells or interfering with PDGFRA signalling in these cells can ameliorate the fibrotic phenotype [126, 127].

## Predisposing factors

A number of risk factors for the development of MPN have been identified. Individuals with CHIP have an enhanced risk of myeloid malignancy [19]. Although these patients are distinguished by the absence of a myeloid neoplasm phenotype, large studies have demonstrated subtle alterations in the full blood counts of these individuals with an increased red cell distribution width and modest increase in white cells and decrease in haemoglobin [117]. More than 75% of mutations in CHIP are accounted for by *ASXL1*, *TET2* and *DNMT3A,* all evident in greater than 5% of MPN cases, whilst the next five most commonly affected genes *JAK2*, *PPM1D*, *SRSF2*, *SF3B1* and *TP53* are all identified at frequencies of around 2% in MPN with the exception of *JAK2,* an MPN driver very commonly identified as discussed [15, 117]. This highlights the similarity between these two groups. Estimates of the prevalence of individuals with detectable *JAK2* V617F mutations falling into the CHIP category suggest significantly more than demonstrate an MPN phenotype [129]. Therefore, the risk of transformation to MPN even with the presence of a driver mutation is not absolute. However, as noted, similarly to the overt MPN group, these *JAK2* V617F positive CHIP individuals have a significantly increased risk of cardiovascular disease [20]. Evidence to base accurate discrimination between patients with clonal haematopoiesis and a low or high risk of transformation to MPN on the basis of genetic or clinical risk factors is less clear than for those with a high risk of leukaemic transformation, and further investigation is required to understand those at risk.

Familial inheritance and germline predisposition to myeloid malignancy has become an important focus of research in the era of widespread genomic analysis [130]. In fact, the heritable risk of MPN is much more significant than evident in many other cancers and higher than for other myeloid malignancy with a ratio of almost 5 observed to expected cases of MPN in individuals with affected first degree relatives [131]. Another study has suggested prevalence of familial cases at approximately 8% [132]. The commonly observed somatic driver mutations in *JAK2* and *CALR* and *MPL* W515L are not inherited. Examples of families with a high penetrance germ line mutation driving thrombocytosis including *MPL* S505N or *MPL* P106L are very rare, but these should be considered in cases with a strong familial components or paediatric patients [133, 134]. Instead, the familial risk appears to result from the presence of predisposing germline susceptibility factors. The *JAK2* 46/1 haplotype has been consistently observed to confer a higher risk of acquiring the *JAK2* V617F. This is true both for *JAK2* V617F positive MPN or CHIP [117, 135]. Similarly, polymorphisms in telomerase reverse transcriptase gene (*TERT*) have been repeatedly identified as independent risk factors for the development of MPN. TERT functions to ensure telomere stability. Several *TERT* polymorphisms have been identified as predisposing factors for MPN and are also associated with the development of other solid tumours [136, 137]. In contrast to dyskeratosis congenita, the *TERT* polymorphisms are associated with telomere lengthening. Increased telomere length has been associated with MPN risk [136]. This extensive genome-wide association study identified a further 15 gene loci in addition to these *TERT* loci increasing risk of MPN. This included loci within *TET2*, *JAK2*, *GATA2*, *ATM*, *RUNX1* and *CHEK2* [136].

Outside of genetic risk factors, the myeloproliferative neoplasms: an in-depth case–control (MOSAICC) study identified a number of potentially modifiable risk factors in the development of MPN. This included childhood household density, low childhood socioeconomic status and a high “pack year” smoking history. Obesity was linked with ET specifically. Alcohol intake was inversely associated with MPN risk [138]. These links may point to a role for inflammation or environmental stressors in modifying the epigenetic risk profile for MPN development. Finally, incidence of PV has traditionally been reported higher in males, whilst the incidence of ET is higher in females. Sex has been observed to be an independent variable for *JAK2 V617F* allele burden with significantly lower allele burdens reported in women compared to men [139].

## Epigenetic dysregulation

Dysregulation of normal epigenetic mechanisms of transcription and translation is increasingly evident in MPN. Activation of the JAK/STAT pathways in response to ligand binding has rapid effect on the chromatin architecture and transcription factor binding profiles [32]. Cytokine-induced changes are different from those induced by chronic constitutive activation of STAT proteins [140]. There is evidence that the histone landscape in MPN is abnormal. These abnormalities can occur even in the absence of any epigenetic modifier mutations. *MPL* W515L mice demonstrate a differential landscape of H3K27 acetylation [85]. Global levels of H3K9 mono and di-methylation are significantly reduced, mediated by enhanced *JMJD1C* expression as a result of *NFE2* overexpression [141]. The mutant JAK2 V617 protein appears able to directly influence the chromatin landscape in a differential manner and independent of STAT protein interactions. JAK2 V617F can interact and phosphorylate protein arginine methyltransferase 5 (PRMT5), impairing PRMT5 histone methylation. This appears to enhance myeloproliferation [142]. JAK2 can locate to the nucleus and mediate phosphorylation of tyrosine 41 on histone H3 (H3Y41). Enhanced levels of H3Y41 were most abundant in *JAK2* V617F containing cell lines. This phosphorylation was reduced by JAK inhibitors and was directly implicated in displacing normal heterochromatin protein 1α [143]. We have recently observed a significant effect of ruxolitinib in the modification of the histone landscape in MPN cell line models and patient samples [84]. In addition, the heterogeneity of histone landscapes will be enhanced by the presence or absence of loss of function mutations in epigenetic modifiers like *EZH2* and *ASXL1*. In *JAK2* V617F mice with conditional *EZH2* deletion, there is a significant downregulation of the transcriptional repressor H3K27me3 mediated by loss of normal polycomb repressive complex 2 function and upregulation of H3K27 acetylation resulting in the activation of genes associated with PMF pathogenesis [144].

DNA methylation has been observed to be abnormal in MPN. There are also differences between disease phenotypes and during progression to blast phase [145, 146]. Studies of particular genes including *SOCS3* and *CD18* have suggested differential methylation status in some MPN patients [81, 147]. Commonly mutated regulators of DNA methylation include *TET2*, *DNMT3A* and *IDH1/2*.

As previously described, components of the spliceosome including *SF3B1*, *SRSF2* and *USAF1* are observed to be mutated in small numbers of MPN patients, particularly those with myelofibrosis. *SF3B1* and *JAK2* mutations are commonly observed together in patients with the distinct overlap syndrome and clinical entity MDS/MPN with ringed sideroblasts and thrombocytosis [148]. Mutant JAK2 V617F has been observed to directly phosphorylate a number of components of the splicing machinery differentially from wild type JAK2 in mouse haematopoietic cells. This results in an alteration in JAK2-ERK signalling to maintain the *JAK2 V617F* clones. JAK2 mutant cells are sensitised to the JAK inhibitor ruxolitinib after inactivation of *YBX1,* a splicing enzyme [59].

## Targeted therapy

As discussed throughout this review, JAK inhibitors, and in particular ruxolitinib, have become a key therapeutic agent in the MPN clinic. They are clearly beneficial in a range of specific MPN patient scenarios with good evidence for spleen volume reduction, haematocrit control and symptom control and some evidence to support a survival benefit in myelofibrosis and with combined hypomethylating agents in blast phase disease [25–27, 149]. Initial hopes of a disease modifying effect similar to tyrosine kinase therapy in CML have not materialised with limited reduction in mutant VAF, limited change in marrow fibrosis and often a limited duration of efficacy prior to loss of response. Exploring the nature of this developed resistance to JAK inhibition in detail is a complex and extensive topic beyond the scope of this article. It is interesting to note that further acquired mutations in JAK2 do not appear to be a significant contributor to resistance in patients. There is evidence to support alternative heterodimer formation between JAK2 and JAK1 or TYK1 in MPN cell line models persistently exposed to ruxolitinib and evidence to support recruitment of alternative MAPK signalling bypassing the JAK/STAT pathway as mechanistic explanations of this resistance [150]. The complexity of the genetic changes within the MPN clone may also determine the responsiveness of the cell to ruxolitinib, whilst new somatic mutations driving clonal evolution on therapy and subsequent expansion of new clones has been clearly documented [151, 152].

There is presently significant interest in targeted combination therapies to augment the beneficial effects of JAK inhibition, particularly in myelofibrosis and blast phase disease. Given the complex features of disease pathogenesis, it is not surprising that a range of therapies targeting intracellular signalling cascades, cytokines and epigenetic regulators have been shown to demonstrate some efficacious features in the management of MPN.

Interferon alpha therapy is increasing recognised as a potential disease modifying agent in MPN and therefore should be classed within the targeted therapeutic approaches. There is clear evidence of reductions in the clonal size and longer term “remissions” of disease are possible [153]. Recent single-cell work demonstrated an enhanced sensitivity to interferon treatment in *JAK2* V617F positive HSCs from ET patients. Treatment appeared to result in the apoptosis of heterozygous cells whilst establishing quiescence in the homozygous cells [125]. In a murine *JAK2* V617F positive model, interferon alpha treatment promoted a shift towards CD41^hi^ expressing HSC population with a megakaryocyte bias and active cell cycling in the *JAK2* V617F positive HSCs, ultimately exhausting the mutant clone [154].

The options for novel therapies in MPN have been recently extensively summarised [155]. Table 2 summarises classes of targeted drugs currently under evaluation in MPN divided into categories based on the sections of this review article.

**Table 2:** Targeted Therapies in MPN

	Drug class	Drug	Approved/trial
JAK-STAT signalling	JAK inhibition	Ruxolitinib	Approved
		Fedratinib	Approved
		Momelotinib	Phase III trial (NCT04173494)
		Pacritinib	Phase III trials (NCT03165734)
Non-JAK/STAT intracellular signalling	PI3K inhibition	Parsaclisib	Phase III (NCT04551066)
	PIM inhibition	PIM447	Phase I (NCT02370706)
Targeted inhibition of mutated proteins	IDH2 inhibition	Enasidenib	Phase II (NCT04281498)
Cell of origin	Interferon-α	Peginterferon-alpha-2A	Approved
		Ropeginterferon-alpha-2B	Approved^a^
Predisposing factors	Telomerase inhibition	Imetelstat	Phase III (NCT04576156)
Epigenetic dysregulation	Hypomethylating agents	Azacitidine	Phase II (NCT01787487)
		Decitabine	Phase II (NCT0428187)
	Histone deacetylase (HDAc) inhibitor	Panobinostat	Phase I/II (NCT01693601)
		Givinostat	Phase II (NCT01761968)
	BET inhibitors	CPI-0610	Phase I/II (NCT02158858)
	LSD1 inhibitors	IMG-7289 (bomedemstat)	Phase II (NCT03136185)
Other	IMiDs	Thalidomide	Phase II (NCT03069326)
	BCL2/BCL-Xl inhibitors	Navitoclax	Phase II (NCT03222609)
	MDM2 inhibition	KRT-232	Phase II (NCT03662126)
	Aurora kinase inhibition	Alisertib	Phase N/A (NCT02530619)
	PD-1 inhibition	Pembrolizumab	Phase II (NCT03065400)
	TGF-beta signalling interference	Luspatercept	Phase II (NCT04717414)
		Sotatercept	Phase II (NCT01712308)
	Anti-CD123	Tagraxofusp	Phase II (NCT02268253)

^a^European Medicines Agency approval only. FDA approval pending

This table summarises the diversity of targeted therapies approved or undergoing clinical trials investigation in MPN either as single agent or combination therapies separated on the basis of sections of this review. The list is not exhaustive and an example of an active or recently completed clinical trial listed on clinicaltrials.gov platform has been provided for each drug. Other trials may be available

## Conclusion

The study of disease provides more than simply a means to alleviating suffering. The insights gained from understanding the molecular pathogenesis of disorders like MPN provide an insight into the phenomenal complexity and simultaneous simplicity with which our cells function. Perhaps most intriguing is the apparent simplicity with which the balance of myeloproliferation is upset and, the relative ease that single mutations in the right cellular context can generate neoplastic clones with an enhanced proliferative drive.

And yet, from the initial discovery of the *JAK2* V617F mutation as a key driver in the majority of MPN patients, it is increasing clear that many more subtleties determine the overall disease phenotype, prognosis and whether “disease” develops at all. The presence of additional pathogenic mutations, the order of acquisition, cellular context, germline predisposition factors, the balance of STAT protein signalling alongside PI3K and MAPK signalling, epigenetic dysregulation and extrinsic influences may all affect the ultimate clonal structure, proliferative drive and differentiation capacity of the neoplastic cells. Genetic complexity and heterogeneity across the population and within single individuals provides a significant diagnostic and therapeutic challenge in MPN. As the medical community transitions into an era in which each stage of work-up and treatment of an individual patient can generate large volumes of information on a scale beyond the analytic capacity of single individuals, we will move further away from discrete classification and categorisation of disease towards individualised clinical, genomic and pathological characterisation. The challenge is how we successfully unleash this potential to understand individualised molecular pathogenesis with translation into effective individualised treatments.

## Data Availability

Not applicable.

## Abbreviations

MPN: Myeloproliferative neoplasm
HSC: Haematopoietic stem cell
PV: Polycythaemia vera
ET: Essential thrombocythaemia
PMF: Primary myelofibrosis
Pre-PMF: Pre-fibrotic primary myelofibrosis
MF: Myelofibrosis
JAK: Janus-associated kinase
STAT: Signal transducer and activator of transcription
WHO: World Health Organisation
CHIP: Clonal haematopoiesis of indeterminate potential
EPO: Erythropoietin
TPO: Thrombopoietin
GCSF: Granulocyte colony stimulating factor
EPOR: Erythropoietin receptor
MPL: Thrombopoietin receptor
GCSFR: Granulocyte colony stimulating factor receptor
CALR: Calreticulin
VAF: Variant allele frequency
SOCS: Suppressor of cytokine signalling
EZH2: Enhancer of Zeste 2
ERK1/2: Extracellular signal related kinase ½
PI3K: Phosphoinositide 3-kinase
MAPK: Mitogen-activated protein kinase
PDGFRα/PDGRFA: Platelet-derived growth factor receptor alpha
mTOR: Mechanistic target of rapamycin
AML: Acute myeloid leukaemia
CML: Chronic myeloid leukaemia
CBL: Cbl proto-oncogene
PTEN: Phosphatase and tension homolog deleted on chromosome 10
DUSP1: Dual-specificity phosphatase 1
NGS: Next-generation sequencing
TERT: Telomerase reverse transcriptase
PRMT5: Protein arginine methyltransferase 5
LSD1: Lysine demethylase 1A
